# Concentration profiles of ions and particles under hydrodynamic focusing in Y-shaped square microchannel

**DOI:** 10.1038/s41598-021-82259-4

**Published:** 2021-01-28

**Authors:** Norikazu Sato, Daisuke Kawashima, Masahiro Takei

**Affiliations:** 1 Sensing & Process Solution Division, JFE Techno-Research Corporation, Kanagawa, 210-0855 Japan; 2 Graduate School of Science and Engineering, Chiba University, Chiba, 263-8522 Japan

**Keywords:** Biomedical engineering, Electrical and electronic engineering, Mechanical engineering

## Abstract

Three-dimensional ion and particle concentrations under hydrodynamic focusing in a Y-shaped square microchannel are numerically simulated to clarify the decrease of the ion concentration along the flow direction within the focused particle stream. The simulation model is theoretically governed by the laminar flow and advection–diffusion equations. The governing equations are solved by the finite volume method. The ion and particle concentration distributions at five cross sections after the confluence of the branch channels are analyzed in 30 cases in which the sheath to sample flow rate ratio *Q*_*sh*_*/Q*_*sam*_ and the Reynolds number *Re* are varied as parameters. The results show that the decrease of the cross-sectional average ion concentration along the flow direction within the particle stream $\overline{c}_{i}$ is described by the diffusion length during the residence time with a characteristic velocity scale. In addition, the deformation of the particle stream due to inertial effects is described by a scaled Reynolds number that is a function of the flow rate ratio. The simulated particle stream thicknesses are validated by theory and a simple experiment. This paper reveals the relationship between the ion and particle concentrations and the dimensionless parameters for hydrodynamic focusing in the Y-shaped square microchannel under typical conditions.

## Introduction

Hydrodynamic focusing in a microfluidic regime is often used to control a sample liquid stream in impedance-based biosensing systems such as the micro-Coulter counter^1^ and conductivity-based biosensor^2^ and in microfluidic impedance cytometry^3^. In these systems, a high-conductivity sample liquid containing ions and cells is focused near the sensing surfaces in a microchannel by coflowing low-conductivity sheath liquids without ions and cells. The high-conductivity focused region confines the electric current in an area near the sensing surfaces in the microchannel, which sensitively increases the electrical impedance when less conductive particles pass through this sensing area. Thus, this flow control technique is effective for enhancing and tuning the impedance sensitivity in a large microchannel^4^.

In order to design and optimize biosensing systems that use hydrodynamic focusing, quantitative evaluation of the mass transport of ions and particles including cells is needed. As a macroscopic approach, the mass transport of ions and particles can be described by the passive scalar transport equations based on advection and diffusion of their concentrations as long as the concentrations are low and other effects including electrical effects are negligible^5^. Advection refers to the transport of a substance by fluid motion, which is determined by the fluid velocity field. Diffusion refers to the net transport of a substance due to random thermal fluctuations, which is determined by the diffusivity of the substance and the spatial gradient of concentration. In biosensing systems that use hydrodynamic focusing, ions and particles are focused near the sensing surface by advection in the confluence region. At the same time, they are transported in the cross-stream direction by diffusion according to the concentration gradient. The extent of diffusion depends on the diffusivity of the cells and ions. For example, in water, the diffusivity of cells, as particles with a size of several microns, is estimated by the Stokes–Einstein equation as *D*_*c*_ ~ 10^–13^ m^2^/s, which is negligibly small in many microfluidic applications, whereas the diffusivity of ions is of the order of *D*_*i*_ ~ 10^–9^ m^2^/s and must not be neglected even at a high Peclet number^3^. This high ion diffusivity causes the noticeable mass transport of ions from the sample liquid to the sheath liquid and leads to a reduction in impedance sensitivity due to a decrease in conductivity around particles in the downstream region. Therefore, prediction of the ion concentration profile along the flow direction within the particle stream is important for electric biosensing systems using hydrodynamic focusing.

Previous studies of the concentration profiles under hydrodynamic focusing in typical Y-shaped rectangular microchannels have separately evaluated the cross-sectional concentration profiles for highly diffusive and less diffusive substances that correspond to ions and particles. Regarding the high-diffusivity substances, the interface broadening caused by cross-stream diffusion was quantified by experiments^6,7^, analytical solutions^8^ and numerical simulations^9,10^ in the case of a sheath to sample flow rate ratio of unity. For less diffusive substances, an analytical expression of the focused stream thickness for various flow rate ratios was proposed by assuming a flat immiscible interface^11^. In addition, the focused stream was found to be deformed due to inertial effects that depends on the confluence angle, flow rate ratio and Reynolds number, which has been demonstrated by experiments and numerical simulations^12^.

However, since these studies did not simultaneously evaluate substances with greatly different diffusivities, such as ions and particles, the ion concentration profile within the particle stream cannot be predicted directly by the methods proposed in the studies. Moreover, these previous studies did not fully explore the parameter space that affects the concentration profiles and did not provide scaling laws that can predict the ion and particle concentration profiles in a wide range of conditions for electric biosensing applications.

In this paper, the three-dimensional ion and particle concentrations under hydrodynamic focusing in a Y-shaped square microchannel are numerically simulated to evaluate the ion concentration profile and particle stream shape. The simulation model predicts the ion and particle concentration distributions at five cross sections in the main channel. A parametric study is performed for two parameters, the sheath to sample flow rate ratio and the Reynolds number. The simulated particle stream thickness is validated by theory and a simple experiment. Finally, scaling analyses for the ion concentration profile and particle stream shape are performed to discuss and clarify the influences of these parameters.

## Methods

### Numerical model

**Figure 1:**
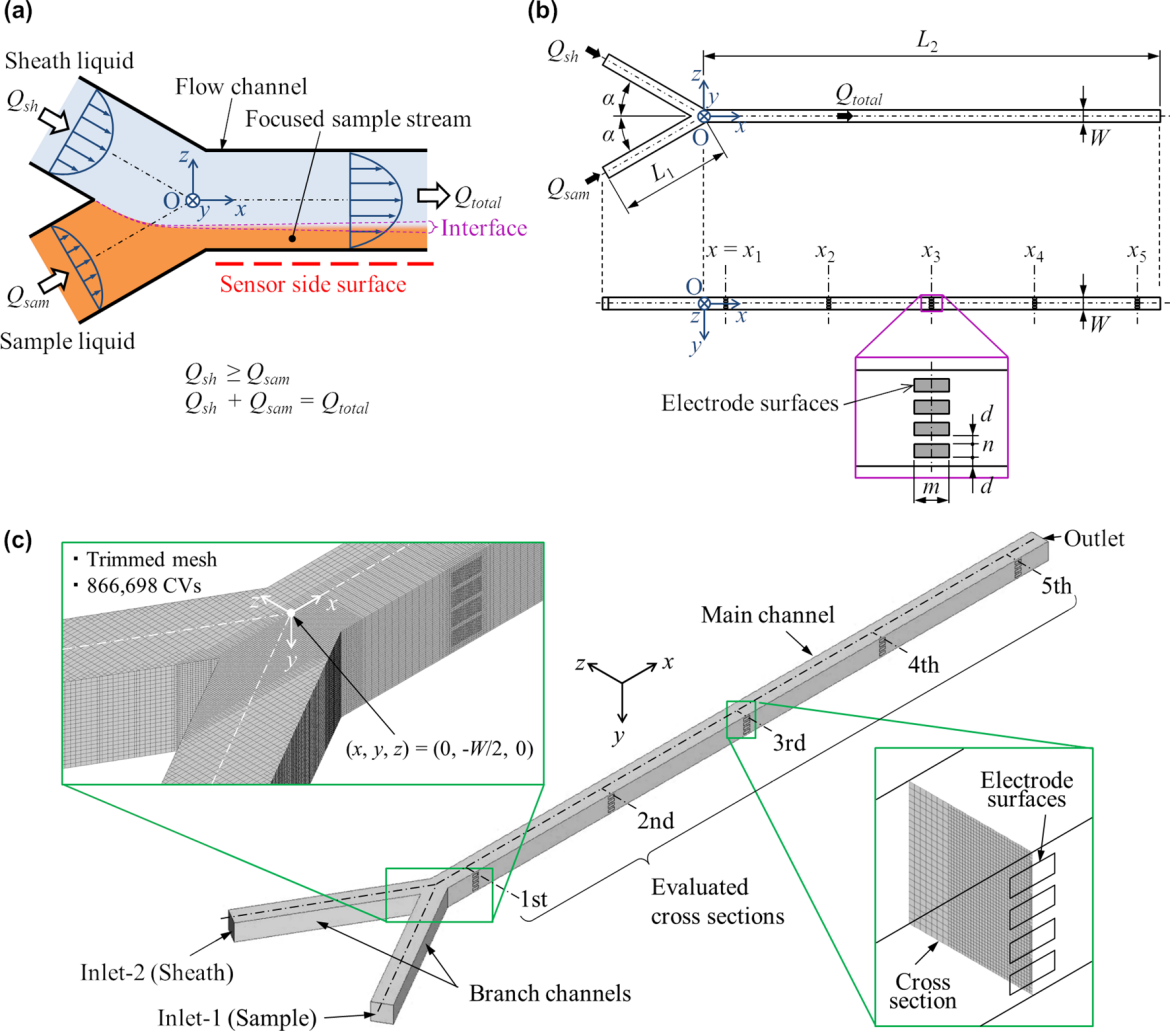
(**a**) Schematic of hydrodynamic focusing in a Y-shaped microchannel. (**b**) Model geometry corresponding to the electrode-multilayered microfluidic device developed by Yao et al.^13,14^. The electrode surface boundaries are prepared at the five longitudinal positions *x* = *x*_1_, *x*_2_, *x*_3_, *x*_4_ and *x*_5_ for future study of electric effects. (**c**) Isometric drawing and computational grid of the model. The insets show the grid resolutions in the confluence region (upper left) and at the cross sections where the ion and particle concentration distributions are evaluated (lower right). The total number of control volumes (CVs) is 866,698.

The fluid flow and mass transports of ions and particles under hydrodynamic focusing in a Y-shaped square microchannel are simulated by using a three-dimensional numerical model. The hydrodynamic focusing in the Y-shaped microchannel is shown schematically in Fig. 1a. The sample liquid is assumed to contain ions and particles with dilute concentrations, whereas the sheath liquid is assumed not to contain them. The simulation model geometry is the symmetric Y-shaped channel with a square cross section shown in Fig. 1b, which is constructed on the basis of the electrode-multilayered microfluidic device developed in the previous work^13,14^. The model dimensions are listed in Table 1. The coordinate system is defined as shown in Fig. 1b. The origin is at the intersection of the center lines of the main and branch channels, as shown in Fig. 1a. The *x* direction is along the flow direction in the main channel. The governing equations of the model are discretized and solved by the finite volume method with the grid resolution shown in Fig. 1c. The base grid size is (Δ*x*, Δ*y*, Δ*z*) = (50 μm, 25 μm, 25 μm). The grids are locally subdivided into Δ*y* = Δ*z* = 12.5 μm for the particle passing region, Δ*x* = Δ*y* = Δ*z* = 12.5 μm near the point of confluence and Δ*x* = Δ*y* = Δ*z* = 6.25 μm near the electrode surface in order to improve spatial resolution, as shown in the inset figures in Fig. 1c. Based on a test comparing the results with twice-finer grids in the *y* and *z* directions, the grid resolution is considered sufficient for the purpose of this study. The test confirmed that the difference in the average ion concentration within the particle stream $\overline{c}_{i}$ in the case of *Re* = 20.7 and *Q*_*sh*_/*Q*_*sam*_ = 30 is less than 4.2% even at the first cross section *x* = *x*_1_, where the difference is considered large.

**Table 1:** Simulation conditions (model dimensions, physical properties and dimensionless parameters).

Model dimensions	Channel width or height *W* [µm]	550
	Channel length *L*_1_, *L*_2_ [mm]	5, 20
	Confluence angle *α* [deg]	30
	Electrode locations in *x*-direction *x*_1_, *x*_2_, *x*_3_, *x*_4_, *x*_5_ [mm]	1, 5.5, 10, 14.5, 19
	Electrode surface dimensions *m*, *n*, *d* [µm]	200, 75, 50
Physical properties	Density *ρ* [kg/m^3^]	1000
	Viscosity *μ* [Pa·s]	0.001
	Dynamic viscosity *ν* (= *μ*/*ρ*) [m^2^/s]	1.0 × 10^–6^
	Diffusivity of ions *D*_*i*_ [m^2^/s]^15^	1.5 × 10^–9^
	Diffusivity of particles *D*_*c*_ [m^2^/s]	~ 10^–13^ ≈ 0
Dimensionless parameters	Sheath to sample flow rate ratio *Q*_*sh*_/*Q*_*sam*_	1, 2, 5, 10, 20, 30
	Reynolds number *Re* = *Q*_*total*_*/*(*Wν*) = (*Q*_*sh*_ + *Q*_*sam*_)*/*(*Wν*)	3.03, 5.56, 10.6, 15.7, 20.7

The sheath and sample liquids in the subject are assumed as miscible fluids with similar densities and viscosities. This assumption means that the fluid flow is unlikely to cause interfacial instability. Accordingly, the flow in the microchannel is considered to be steady-state unless the inlet flow condition varies with time. In addition, the particles are assumed to be several microns in size and neutrally buoyant in the fluid, which is not affected by gravitational force. These assumptions can be applied when cells are suspended in an aqueous solution with a flow in typical biosensing. For ion concentration, gravity effects are not considered because the ions are dissolved in water. Since the particles in this simulation are considered to move on the streamline because of their very small Stokes number and negligible lateral motion (due to diffusion, lift force, gravitational force, etc.), the particle concentration within the particle stream is assumed to be constant.

The fluid flow in the microchannel in the steady-state is modeled by the incompressible Navier–Stokes equation and the continuity equation. Assuming that the density and viscosity of the sample and sheath fluids are the same, the steady-state fluid flow is governed by:

1 $$ \rho ({\mathbf{u}} \cdot \nabla ){\mathbf{u}} = - \nabla p + \mu \,\nabla^{2} {\mathbf{u}}, $$

2 $$ \nabla \cdot {\mathbf{u}} = 0, $$

where **u**, *p*, *μ* and *ρ* are the velocity, pressure, density and viscosity of the fluid. In the absence of an electric field, the macroscopic mass transports of ions and particles in dilute solutions are described as passive scalars obeying the following advection diffusion equations:

3 $$ {\mathbf{u}} \cdot \nabla c_{i} = D_{i} \nabla^{2} c_{i}, $$

4 $$ {\mathbf{u}} \cdot \nabla c_{c} = D_{c} \nabla^{2} c_{c}, $$

where *c*_*i*_ and *c*_*c*_ are the normalized ion and particle concentrations, and *D*_*i*_ and *D*_*c*_ are the diffusivities of the ions and particles. When the diffusivity of particles *D*_*c*_ is negligibly small, Eq. (4) is reduced to the simple advection equation:

5 $$ {\mathbf{u}} \cdot \nabla c_{c} = 0. $$

The simulation conditions are listed in Table 1. The fluid is assumed to have a constant density and viscosity corresponding to those properties of water. *D*_*i*_ is assumed to be that of NaCl in water^15^. *D*_*c*_ is assumed to be zero because *D*_*c*_ is negligibly small according to the evaluation by the Stokes–Einstein correlation for particles with a size of several microns and is considered to be low compared to the numerical diffusivity in this model. A parameter study is performed with a total of 30 cases in which the two dimensionless parameters sheath to sample flow rate ratio *Q*_*sh*_/*Q*_*sam*_ and Reynolds number *Re* are varied as shown in Table 1.

The boundary conditions are summarized in Table 2. For the fluid flow, the velocity **u** is prescribed at the inlets and channel wall. The pressure *p* is prescribed at the outlet. For the ion and particle concentrations, the normalized concentrations *c*_*i*_ and *c*_*c*_ are prescribed at the inlets, and their gradients are prescribed at the other boundaries. Equations (1) and (2) governing the fluid flow are solved by the SIMPLE method^16^. Then, Eqs. (3) and (5) are solved independently using the fluid velocity. According to the above-mentioned zero diffusivity assumption, the particle concentration should vary sharply at the boundary of the particle passing region. To capture the sharp variation of concentration with a finite grid resolution, special discretization for the advection term in Eq. (5) is needed in order to avoid excessive spreading due to numerical diffusion. This model uses a high resolution advection scheme based on the HRIC scheme^17^ for Eq. (5) to reduce numerical diffusion. The HRIC scheme is widely used to capture sharp interfaces between immiscible fluids.

**Table 2:** Summary of the boundary conditions. Where **n** is the unit outward normal vector on the boundary of the model. The sample and sheath inlet velocities are prescribed according to the simulation parameters *Re* and *Q*_*sh*_*/Q*_*sam*_ shown in Table 1.

Boundary	Fluid flow	Ion and particle concentrations
Sample inlet (Inlet-1)	${\mathbf{u}} \cdot {\mathbf{n}} = - {{Q_{sam} } \mathord{\left/ {\vphantom {{Q_{sam} } {W^{2} }}} \right. \kern-\nulldelimiterspace} {W^{2} }}$, ${{Q_{sam} = ReW\nu } \mathord{\left/ {\vphantom {{Q_{sam} = ReW\nu } {\left( {1 + {{Q_{sh} } \mathord{\left/ {\vphantom {{Q_{sh} } {Q_{sam} }}} \right. \kern-\nulldelimiterspace} {Q_{sam} }}} \right)}}} \right. \kern-\nulldelimiterspace} {\left( {1 + {{Q_{sh} } \mathord{\left/ {\vphantom {{Q_{sh} } {Q_{sam} }}} \right. \kern-\nulldelimiterspace} {Q_{sam} }}} \right)}}$	*c*_*i*_ = *c*_*c*_ = 1
Sheath inlet (Inlet-2)	${\mathbf{u}} \cdot {\mathbf{n}} = - {{Q_{sh} } \mathord{\left/ {\vphantom {{Q_{sh} } {W^{2} }}} \right. \kern-\nulldelimiterspace} {W^{2} }}$, ${{Q_{sh} = ReW\nu } \mathord{\left/ {\vphantom {{Q_{sh} = ReW\nu } {\left( {1 + {{Q_{sam} } \mathord{\left/ {\vphantom {{Q_{sam} } {Q_{sh} }}} \right. \kern-\nulldelimiterspace} {Q_{sh} }}} \right)}}} \right. \kern-\nulldelimiterspace} {\left( {1 + {{Q_{sam} } \mathord{\left/ {\vphantom {{Q_{sam} } {Q_{sh} }}} \right. \kern-\nulldelimiterspace} {Q_{sh} }}} \right)}}$	*c*_*i*_ = *c*_*c*_ = 0
Outlet	*p* = 0	$\nabla c_{i} \cdot {\mathbf{n}} = \nabla c_{c} \cdot {\mathbf{n}} = 0$
Channel wall	**u** = **0**	$\nabla c_{i} \cdot {\mathbf{n}} = \nabla c_{c} \cdot {\mathbf{n}} = 0$

### Theoretical expression of particle stream thickness

A theoretical expression of the particle stream thickness under hydrodynamic focusing is derived to evaluate and to discuss the particle stream shape. The focused particle stream thickness in a rectangular channel can be predicted from the velocity profile equation by assuming the flat immiscible interface. From the analytical solution for a rectangular channel^18^, the velocity profile of the developed flow for a square channel is expressed by

6 $$ u\left( {y,z} \right) = \frac{{4W^{2} }}{{\mu \pi^{3} }}\left( { - \frac{{{\text{d}}p}}{{{\text{d}}x}}} \right)\sum\limits_{i = 1,3,5, \ldots }^{\infty } {\left( { - 1} \right)^{(i - 1)/2} \left[ {1 - \frac{{\cosh \left( {{{i\pi z} \mathord{\left/ {\vphantom {{i\pi z} W}} \right. \kern-\nulldelimiterspace} W}} \right)}}{{\cosh \left( {{{i\pi } \mathord{\left/ {\vphantom {{i\pi } 2}} \right. \kern-\nulldelimiterspace} 2}} \right)}}} \right]} \frac{{\cos \left( {{{i\pi y} \mathord{\left/ {\vphantom {{i\pi y} W}} \right. \kern-\nulldelimiterspace} W}} \right)}}{{i^{3} }}, $$

where *u* is the *x*-component of the velocity vector. Similar to Lee et al.^11^, the average velocity along the *y* direction is derived by integrating Eq. (6) as

7 $$ \overline{u}\left( z \right) = \frac{1}{W}\int_{{ - {W \mathord{\left/ {\vphantom {W 2}} \right. \kern-\nulldelimiterspace} 2}}}^{{{W \mathord{\left/ {\vphantom {W 2}} \right. \kern-\nulldelimiterspace} 2}}} {u\left( {y,z} \right)} \,{\text{d}}y\,\, = \,\,\frac{{8W^{2} }}{{\mu \pi^{4} }}\left( { - \frac{{{\text{d}}p}}{{{\text{d}}x}}} \right)\sum\limits_{i = 1,3,5, \ldots }^{\infty } {\frac{1}{{i^{4} }}\left[ {1 - \frac{{\cosh \left( {{{i\pi z} \mathord{\left/ {\vphantom {{i\pi z} W}} \right. \kern-\nulldelimiterspace} W}} \right)}}{{\cosh \left( {{{i\pi } \mathord{\left/ {\vphantom {{i\pi } 2}} \right. \kern-\nulldelimiterspace} 2}} \right)}}} \right]}. $$

Then, the flow rate ratio *Q*_*sam*_*/Q*_*total*_ is derived by integrating Eq. (7) along the *z* direction as

8 $$ \frac{{Q_{sam} }}{{Q_{total} }} = \frac{{\int_{ - W/2}^{{\delta - {W \mathord{\left/ {\vphantom {W 2}} \right. \kern-\nulldelimiterspace} 2}}} {\overline{u}\left( z \right){\text{d}}z} }}{{\int_{ - W/2}^{{ - {W \mathord{\left/ {\vphantom {W 2}} \right. \kern-\nulldelimiterspace} 2}}} {\overline{u}\left( z \right){\text{d}}z} }} = f\left( {\frac{\delta }{W}} \right) = \frac{{\frac{\delta }{W} - \frac{96}{{\pi^{5} }}\sum\limits_{i = 1,3,5, \ldots }^{\infty } {\frac{{\sinh \left( {{{i\pi \delta } \mathord{\left/ {\vphantom {{i\pi \delta } W}} \right. \kern-\nulldelimiterspace} W} - {{i\pi } \mathord{\left/ {\vphantom {{i\pi } 2}} \right. \kern-\nulldelimiterspace} 2}} \right) + \sinh \left( {{{i\pi } \mathord{\left/ {\vphantom {{i\pi } 2}} \right. \kern-\nulldelimiterspace} 2}} \right)}}{{i^{5} \cosh \left( {{{i\pi } \mathord{\left/ {\vphantom {{i\pi } 2}} \right. \kern-\nulldelimiterspace} 2}} \right)}}} }}{{1 - \frac{192}{{\pi^{5} }}\sum\limits_{i = 1,3,5, \ldots }^{\infty } {\frac{{\tanh \left( {{{i\pi } \mathord{\left/ {\vphantom {{i\pi } 2}} \right. \kern-\nulldelimiterspace} 2}} \right)}}{{i^{5} }}} }}, $$

where *δ* is the focused layer thickness assuming the flat immiscible interface. Equation (8) gives the relationship between the flow rate ratio *Q*_*sam*_*/Q*_*total*_ and the scaled focused layer thickness *δ/W*.

### Experiment

**Figure 2:**
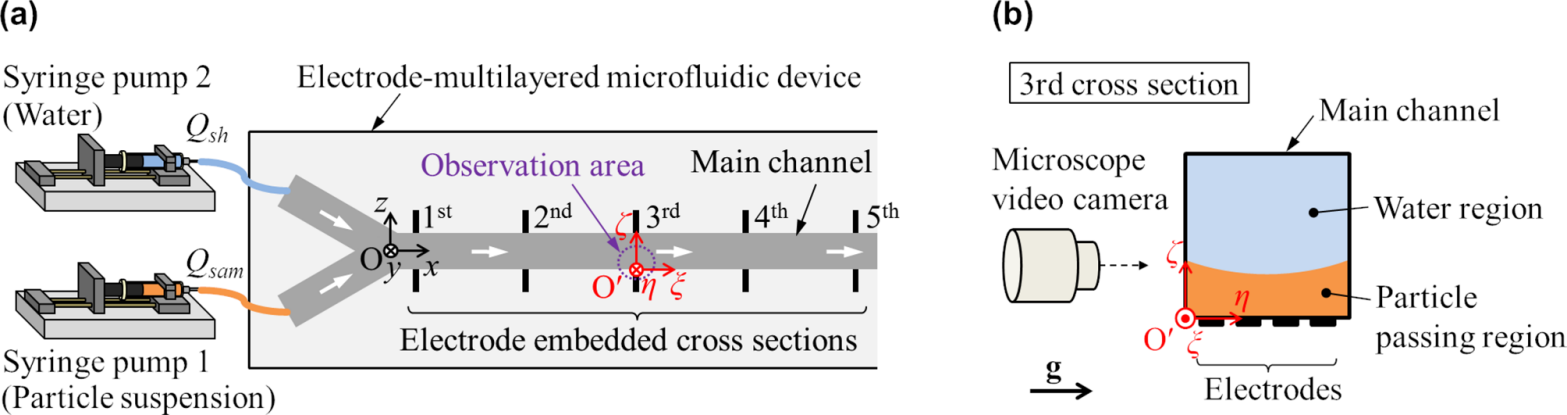
(**a**) Experimental setup. The particle suspension as the sample liquid and water as the sheath liquid were injected into the device by two syringe pumps with different flow rate ratios *Q*_*sh*_/*Q*_*sam*_. (**b**) Schematic of the particle stream observation method. A local coordinate system with the origin at the third cross section is defined in order to evaluate the particle stream thickness.

A simple experiment was conducted to validate the simulated particle stream thickness under hydrodynamic focusing. Figure 2 shows the experimental setup, which consists of the electrode-multilayered microfluidic device, two syringe pumps (KDS100; Muromachi Kikai Co., Ltd., Japan), a microscope (Eclipse LV 100; Nikon, Japan) and a high speed camera (Fastcam SA3; Photron, Japan). The microfluidic device was fabricated by Yao et al. Since the fabrication details were described in the paper by Yao et al.^13^, only the main procedures are summarized here, as follows: (1) Quartz substrates with electrodes made by platinum vapor deposition are layered and compressed to create four electrode layers within five substrates. (2) The layered substrates are drilled to form a Y-shaped microchannel, which is the subject in this study. (3) The drilled substrates are placed between additional quartz substrates to form top and bottom walls of the microchannel. (4) Inlet and outlet holes and copper terminals are provided.

The experiment was conducted using the Y-shaped microchannel of the electrode-multilayered microfluidic device. The sample liquid was obtained by suspending a particle pigment having a diameter of 3 ± 1 µm in water with a concentration of approximately 0.1 vol%. The sheath liquid was water. The sample and sheath liquids were injected into the respective branch channels of the device at constant flow rates using two syringe pumps. Microscopic images at the third cross section were taken while the particles and fluids were passing through the channel, as shown in Fig. 2b.

## Results

### Concentration distribution

**Figure 3:**
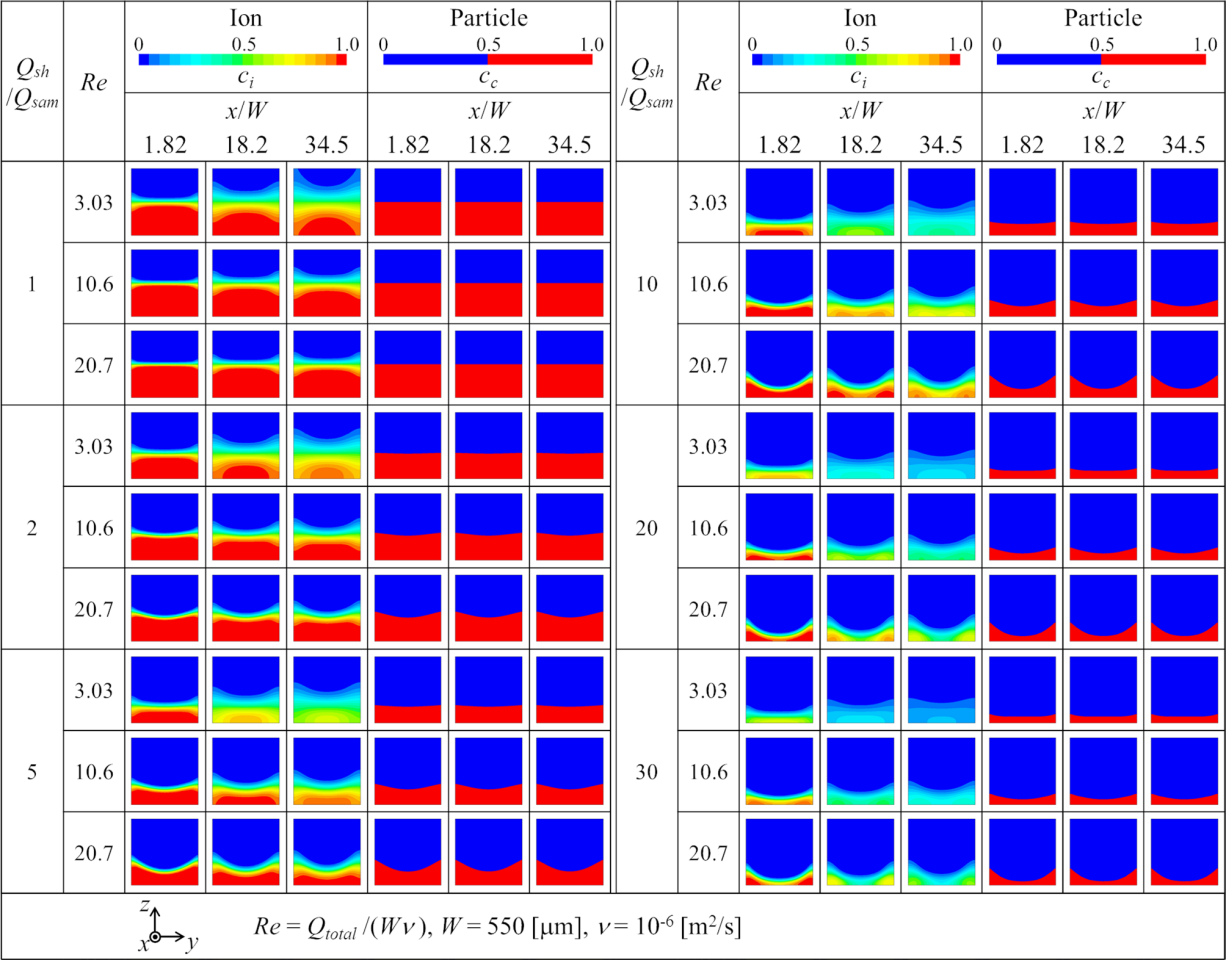
Normalized concentration distributions of ions and particles at the three cross sections in the main flow channel for the three Reynolds numbers.

The three-dimensional fluid flow and the ion and particle concentrations are simulated to obtain the concentration distributions at the five cross sections *x* = *x*_1_, *x*_2_, *x*_3_, *x*_4_ and *x*_5_ shown in Fig. 1b. Figure 3 shows the normalized concentration distributions of the ions and particles at the three cross sections *x* = *x*_1_, *x*_3_ and *x*_5_ in the main channel for the three Reynolds numbers *Re* = *Q*_*total*_*/*(*Wν*) = 3.03, 10.6 and 20.7. The ion concentration is displayed by the filled contour with 20 color levels, whereas the particle concentration is displayed by the binary image colored blue for the particle non-existence area and red for the particle existence area because of the zero diffusivity assumption for the particles. According to Fig. 3, the particle stream is focused more thinly on the sensor side wall (*z* =  − *W/*2) as *Q*_*sh*_*/Q*_*sam*_ increases. In addition, the focused particle stream layer is deformed to be thinner near the center line (*y* = 0) and thicker near the side walls (*y* =  ± *W/*2) as the Reynolds number increases. Although the ion concentration is also focused and deformed similarly to the particle stream, the interface is blurred due to diffusion as the fluid flows downstream. The diffuse interface width where the ion concentration changes in the *z* direction is wider near the side wall (*y* =  ± *W/*2) than near the center line (*y* = 0). This diffuse concentration pattern, known as the butterfly effect^6^, is due to the residence time variation caused by the velocity profile in the channel. The diffuse interface width decreases as the Reynolds number increases.

### Ion concentration within particle stream

**Figure 4:**
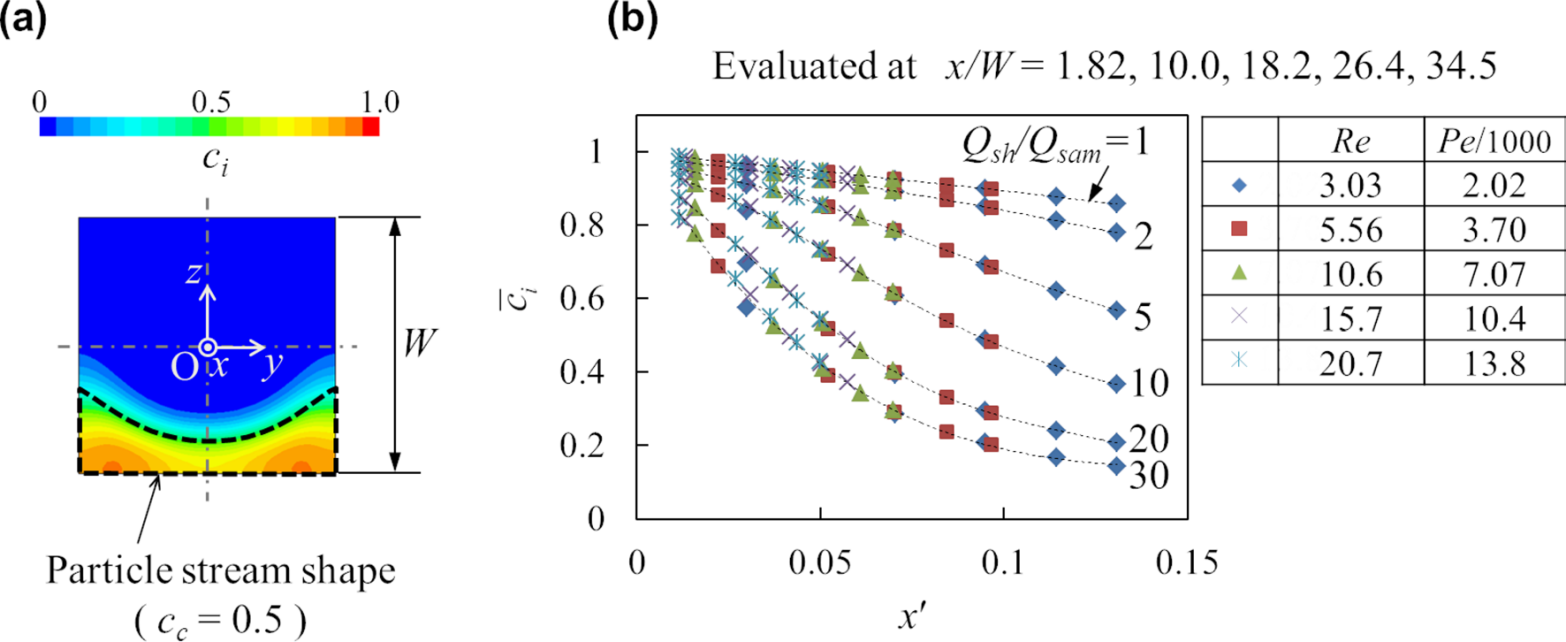
Decrease of the ion concentration along the flow direction within the particle stream. (**a**) An example of the ion concentration contour at a cross section. The dash line shows the particle stream shape in which the average ion concentration is evaluated. (**b**) Change of the average ion concentration $\overline{c}_{i}$ along the dimensionless length *x*′ = [(*x/W*)*/Pe*]^1/2^. The dotted lines are polynomial fits to show the connection of the data for each flow rate ratio.

The decrease of the ion concentration within the particle stream along the flow direction is quantified from the concentration distributions at the cross sections. In order to evaluate the ion concentration within the particle stream shown by the dash line in Fig. 4a, the average concentration $\overline{c}_{i}$ is defined as

9 $$ \overline{c}_{i} = \frac{{\int_{A} {c_{c} c_{i} \,{\text{d}}A} }}{{\int_{A} {c_{c} \,{\text{d}}A} }}, $$

where *A* is the cross-sectional area. In addition, the dimensionless length considering diffusion *x*′is defined as

10 $$ x^{\prime} = \sqrt {{{\left( \frac{x}{W} \right)} \mathord{\left/ {\vphantom {{\left( \frac{x}{W} \right)} {Pe}}} \right. \kern-\nulldelimiterspace} {Pe}}} = \frac{1}{W}\sqrt {\frac{{D_{i} x}}{U}} , $$

where *Pe* = *UW/D*_*i*_ = *Q*_*total*_*/*(*WD*_*i*_) is the Peclet number and *U* = *Q*_*total*_*/W*^2^ is the cross-sectional mean velocity in the main channel. As shown on the right side of Eq. (10), the dimensionless length *x*′ is regarded as the diffusion length during the mean residence time after confluence (*D*_*i*_* x/U*)^1/2^ scaled by the channel size *W*. Figure 4b shows the relationship between the average ion concentration $\overline{c}_{i}$ and the dimensionless length *x*′. According to Fig. 4b, the average ion concentration $\overline{c}_{i}$ decreases along the flow direction as *Q*_*sh*_*/Q*_*sam*_ increases and is clearly described by one curve for each *Q*_*sh*_*/Q*_*sam*_ regardless of the Peclet or Reynolds numbers.

### Shape of particle stream

**Figure 5:**
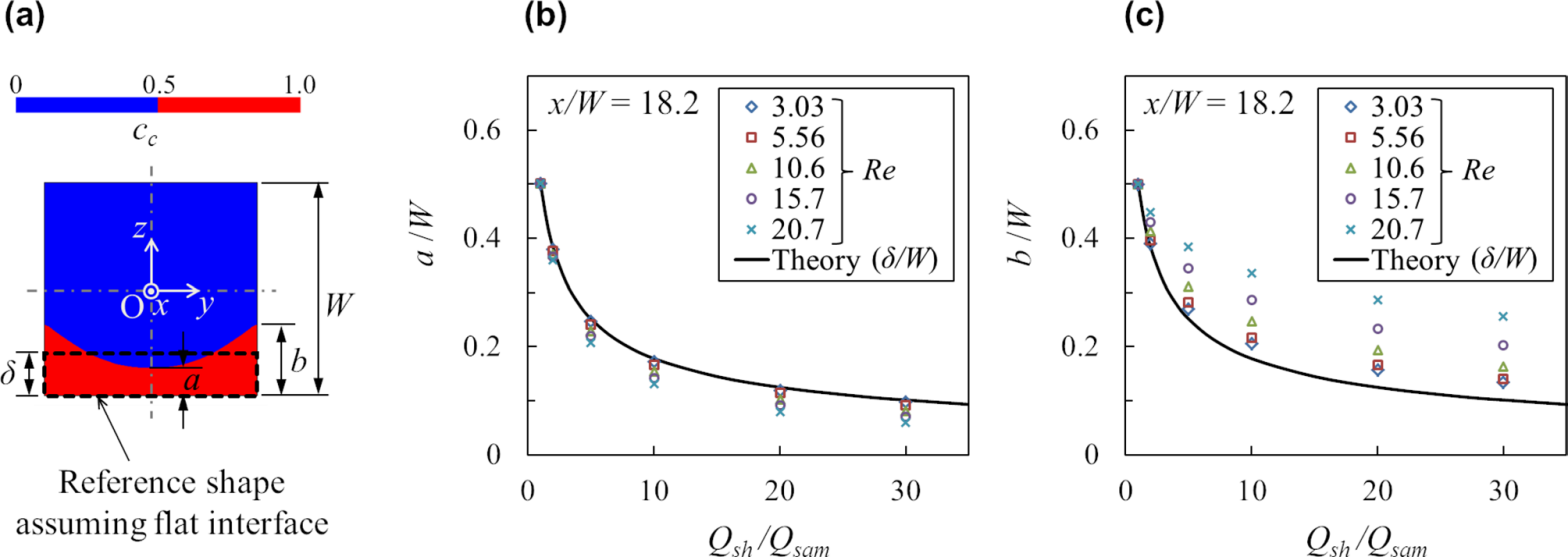
Cross-sectional shape of the particle stream. (**a**) An example of the particle concentration distribution at a cross section and description of the symbols for each part of the thickness of the particle stream. The dash line indicates the theoretical particle stream shape assuming the flat immiscible interface. (**b**,**c**) Changes of the thicknesses at the center and on the side wall, *a/W* and *b/W*, with respect to the flow rate ratio *Q*_*sh*_*/Q*_*sam*_. The solid line is the theoretical particle stream thickness calculated by Eq. (8).

The particle stream shape is quantified from the particle existence area recognized from the above-mentioned particle concentration distributions at the cross sections. According to Fig. 3, since the particle concentration shape is regarded as unchanged in the *x* direction, the two thicknesses of the focused layer *a* and *b* illustrated in Fig. 5a are determined from the points where *c*_*c*_ is interpolated as 0.5 from the *c*_*c*_ values at the discrete calculated points on the center line (*y* = 0) and on the side wall (*y* =  ± *W/*2) at the third cross section of *x/W* = 18.2. Figure 5b,c show the changes of the particle stream thicknesses at the center and on the side wall, *a/W* and *b/W*, with respect to the flow ratio *Q*_*sh*_*/Q*_*sam*_, with the Reynolds number as a parameter. As a reference, these figures also show the theoretical thickness *δ/W* when the flat immiscible interface is assumed, which is calculated by Eq. (8). According to Fig. 5b,c, both thicknesses *a/W* and *b/W* decrease as *Q*_*sh*_*/Q*_*sam*_ increases due to hydrodynamic focusing. In the cases of *Q*_*sh*_*/Q*_*sam*_ = 1, the thicknesses are constant at *a/W* = *b/W* = 0.5 regardless of the Reynolds number. In the cases of *Q*_*sh*_*/Q*_*sam*_ > 1, *a/W* is thinner than *δ/W* and becomes thinner as the Reynolds number increases. In contrast, *b/W* is thicker than *δ/W* and becomes thicker as the Reynolds number increases.

### Experimental validation

**Figure 6:**
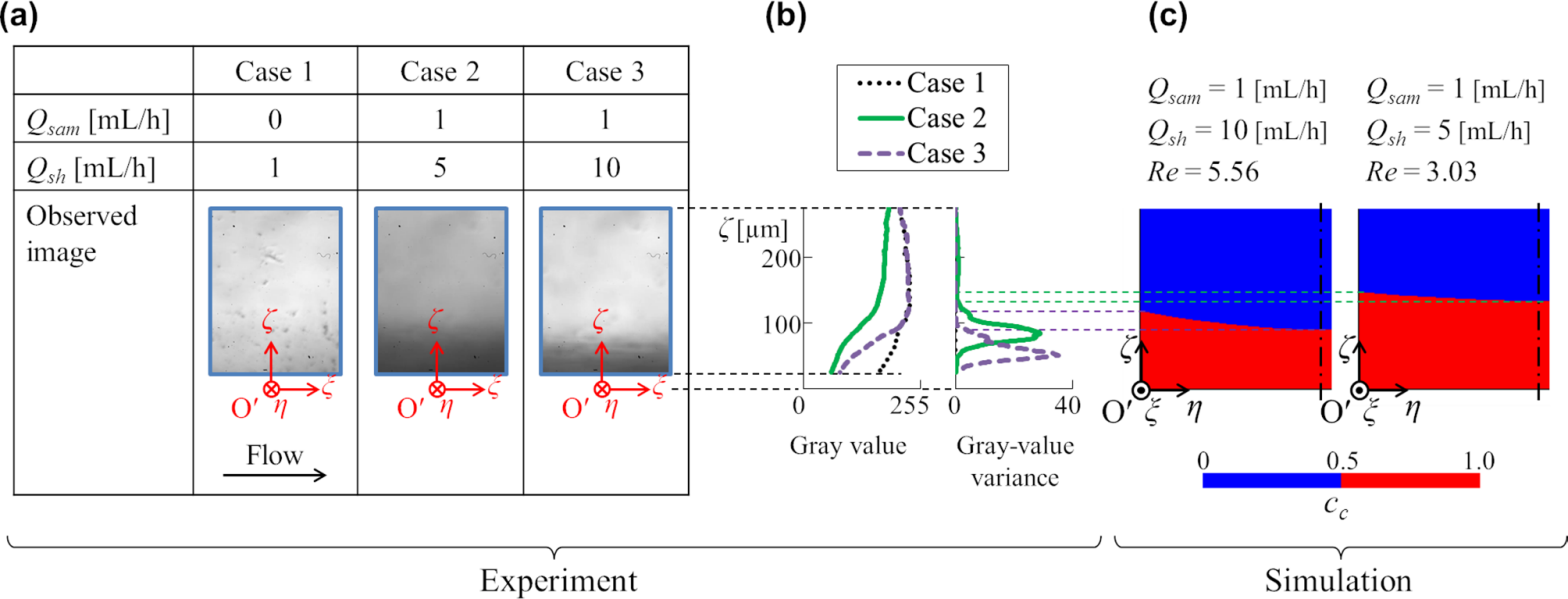
Comparison between experimental and simulated particle stream thicknesses. (**a**) Observation images for three cases of different sample and sheath flow rates. (**b**) *ζ*-direction distributions of the *ξ*-directional average gray value and its time variance of the images in (**a**). (**c**) Simulated particle concentration distributions at the third cross section under conditions corresponding to Case 2 (right) and Case 3 (left) in the experiment.

The simulated particle stream thickness is validated by the simple experiment as shown in Fig. 2. Figure 6 shows a comparison between the experimental and simulated focused layer thicknesses. Here, 150 images were taken at 100 fps for three cases: Case 1, 2 and 3 in Fig. 6a. Then, the pixel gray values (0–255) of the images were analyzed to identify the particle existence area as the focused layer. Figure 6a shows the first frames of the images for the three cases. Figure 6b shows the *ζ*-direction profiles of the *ξ*-directional average gray value and their time variance of the images. Figure 6c shows the simulated particle concentration distributions that correspond to Case 2 and Case 3 in Fig. 6a. According to Fig. 6a–c, although the correspondence between the gray values and the layer thicknesses is unclear, the largest ζ positions where the time variances are prominent (thin dotted lines over Fig. 6b,c) agree with the projected interface positions in the experiment. This is because the time variance is mainly caused by the passage of the particles. The projected interface positions in this experiment are reasonably consistent with those in the simulation.

## Discussion

Scaling analyses for the ion concentration profile and the particle stream shape are performed in order to further clarify the influences of the two dimensionless parameters: the flow rate ratio *Q*_*sh*_*/Q*_*sam*_ and the Reynolds number *Re* (Peclet number *Pe*). Note that the Peclet number is described by *Pe* = *Sc Re* and cannot be changed independently from *Re* without changing the fluid (or the Schmidt number *Sc* = *v/D*_*i*_).

**Figure 7:**
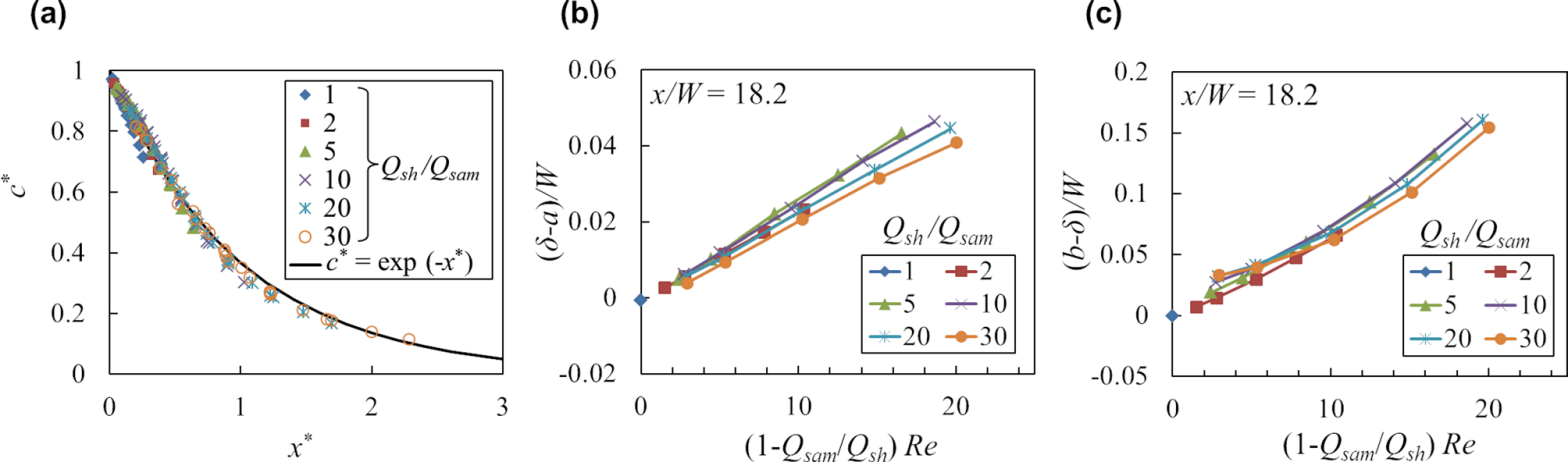
Scaling analyses of the ion concentration profile and particle stream deformation. (**a**) Relationship between the normalized concentration change c^*^ described by Eq. (11) and the rescaled dimensionless length x^*^ described by Eq. (12). (**b**,**c**) Concave depth (*δ*-*a*)*/W* and cusp height (*b*-*δ*)*/W* of the particle stream as functions of the scaled Reynolds number (1-*Q*_*sam*_*/Q*_*sh*_)*Re*.

Regarding the ion concentration profile, all the curves in Fig. 4b can be approximately reduced to one curve as shown in Fig. 7a by taking the following normalized ion concentration change c^*^ and dimensionless length x^*^ rescaled using the theoretical particle stream thickness *δ*.

11 $$ c^{*} = \frac{{\overline{c}_{i} - \left( {{{Q_{sam} } \mathord{\left/ {\vphantom {{Q_{sam} } {Q_{total} }}} \right. \kern-\nulldelimiterspace} {Q_{total} }}} \right)}}{{1 - \left( {{{Q_{sam} } \mathord{\left/ {\vphantom {{Q_{sam} } {Q_{total} }}} \right. \kern-\nulldelimiterspace} {Q_{total} }}} \right)}}, $$

12 $$ x^{*} = \sqrt {\frac{{\left( {1 + {{Q_{sh} } \mathord{\left/ {\vphantom {{Q_{sh} } {Q_{sam} }}} \right. \kern-\nulldelimiterspace} {Q_{sam} }}} \right)\left( {{x \mathord{\left/ {\vphantom {x \delta }} \right. \kern-\nulldelimiterspace} \delta }} \right)}}{Pe}} = \frac{1}{\delta }\sqrt {\frac{{D_{i} x}}{{{{Q_{sam} } \mathord{\left/ {\vphantom {{Q_{sam} } {\left( {W\delta } \right)}}} \right. \kern-\nulldelimiterspace} {\left( {W\delta } \right)}}}}} , $$

where (*Q*_*sam*_*/Q*_*total*_) means the concentration of *c*_*i*_ under complete mixing, which assumes a well-mixed state with a concentration gradient of zero. This scaling shows that the normalized concentration change c^*^ is approximately described by a function of the dimensionless diffusion length x^*^ during the residence time with the characteristic velocity *U*_*a*_ = *Q*_*sam*_*/*(*Wδ*), which is the mean velocity within the particle stream under the assumption of the flat immiscible interface. Although the particle stream deforms due to the inertial effect in the high Reynolds number region, this scaling uses the characteristic velocity under the assumption of the flat interface. This scaling data can be fitted by a simple function of c^*^ = exp (−x^*^) within 5.6% error of full scale, as shown in Fig. 7a.

Regarding the particle stream shape, the thickness differences from the flat interface *δ*−*a* and *b*−*δ* read from Fig. 5b,c are considered to be due to the inertial effect demonstrated by Nasir et al.^12^. Without the inertial effect as *Re* ≈ 0, the particle stream shape is considered to be flat and its thickness *δ* is predicted by Eq. (8). Figure 7b,c show the dimensionless concave depth and cusp height from the flat interface (*δ*−*a*)*/W* and (*b*−*δ*)*/W* with respect to the dimensionless number (1 − *Q*_*sam*_*/Q*_*sh*_)*Re*. According to these figures, (*δ*−*a*)*/W* and (*b*−*δ*)*/W* are found to be described by the Reynolds number scaled by a simple function of the flow rate ratio as (1 − *Q*_*sam*_*/Q*_*sh*_). The cusp height (*b*−*δ*)*/W* range is found to be three or four times larger than that of the concave depth (*δ*−*a*)*/W*. This scaling is also consistent with the cross-sectional stream shape reported by Nasir et al.^12^.

From the above discussion, the average ion concentration profile and the particle stream deformation with the Reynolds number are found to be scaled with relatively simple functions by using the theoretical particle stream thickness *δ*. These relationships among the dimensionless numbers are expected to be useful for design or evaluation of the hydrodynamic focusing in a Y-shaped square channel over a wide range of conditions in many microfluidic applications.

## Conclusions

This study numerically revealed the ion concentration profile and particle stream shape under hydrodynamic focusing in the Y-shaped square microchannel in the ranges of the flow rate ratio *Q*_*sh*_*/Q*_*sam*_ = 1 to 30 and the Reynolds number *Re* ≈ 3.0 to 21, which corresponds to the Peclet number range of *Pe* ≈ 2.0 × 10^3^ to 1.4 × 10^4^.

The decrease of the cross-sectional average ion concentration along the flow direction within the particle stream, which changes depending on the flow rate ratio and Reynolds number, was evaluated from the concentration distribution simulated by the three-dimensional numerical model. The simulated particle stream thickness was consistent with the theoretical expression and the simple observation experiment.

The scaling analyses based on the simulated results showed that the change in the average ion concentration in the flow direction was scaled by the diffusion length during the residence time with the average velocity of the particle stream. In addition, the change of the particle stream thickness due to inertial effects was scaled by a simple function of the flow rate ratio and Reynolds number.
